# Supplementary material for: Eph Receptor Tyrosine Kinases Are Functional Entry Receptors for Murine Gammaherpesvirus 68
Source: bioRxiv. 2025 Jun 5:2025.06.05.657996. Preprint. [Version 1] doi: 10.1101/2025.06.05.657996 (PMC12157678; doi:10.1101/2025.06.05.657996)
Supplement: Supplement 1 [file NIHPP2025.06.05.657996v1-supplement-1.pdf]

bioRxiv preprint doi: https://doi.org/10.1101/2025.06.05.657996; this version posted June 5, 2025. The copyright holder for this preprint (which was not certified by peer review) is the author/funder. This article is a US Government work. It is not subject to copyright under 17 USC 105 and is also made available for use under a CC0 license.

S1 Fig

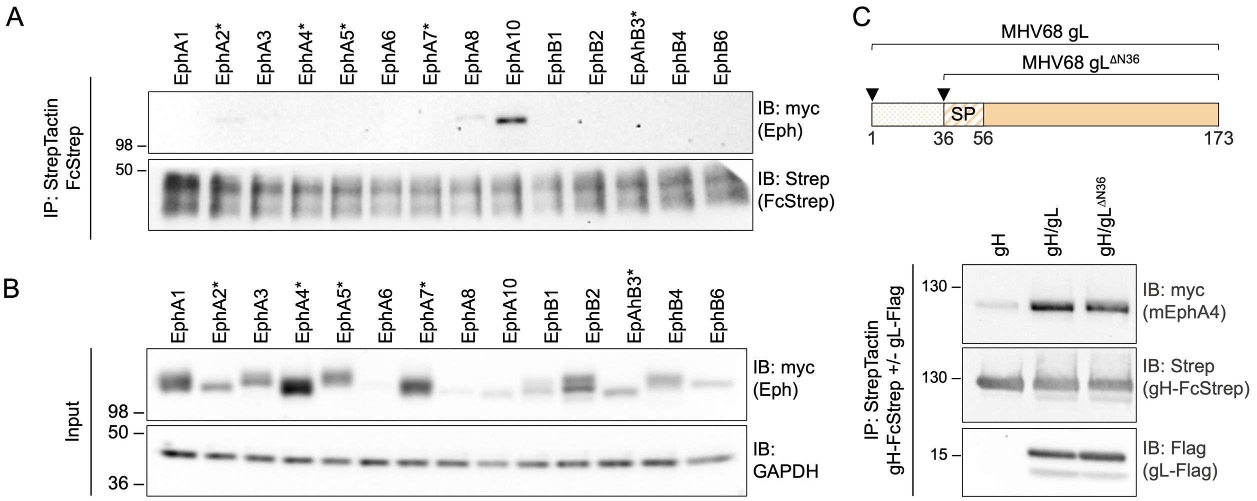

D

	Accession number		
	human	mouse	Identity
**EphA1**	AAI30292.1*	NP_076069.2	88.36%
**EphA2**	P29317*	NP_034269.2†	92.23%
**EphA3**	AAH63282*	NP_034270.1	96.34%
**EphA4**	NP_004429*	NP_031962.2†	98.58%
**EphA5**	NP_004430*	NP_001389678.1	93.85%
**EphA6**	NP_001073917*	NP_031964.2†	93.85%
**EphA7**	AAI43858*	NP_034271.3†	95.05%
**EphA8**	AAI41437*	NP_031965.2	95.22%
**EphA10**	NP_001092909*	NP_001243361.1	92.66%

	Accession number		
	human	mouse	Identity
**EphB1**	AAI11745*	NP_775623.2†	98.58%
**EphB2**	NP_059145*	NP_001277682.1	99.39%
**EphB3**	NP_004434*	NP_034273.1†	96.00%
**EphB4**	AAH52804*	NP_034274.4	93.01%
**EphB6**	NP_004436*	NP_031706.3	91.79%

**Amino acid sequences identical to human Eph expression constructs used in this study.*
**Amino acid sequences identical to murine Eph expression constructs used in this study.*

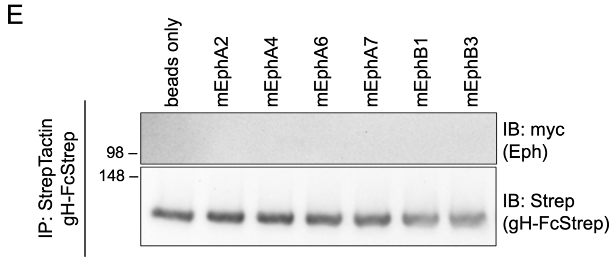

S1 Fig. The MHV68 gH/gL complex binds human and murine Eph receptors.

(A) Pairwise precipitation of soluble recombinant FcStrep with individual human Eph proteins. Precipitates were analyzed by immunoblot with indicated antibodies. Asterisks indicate known KSHV, EBV or RRV gH/gL interaction partners. **(B)** Input immunoblot for individual human Eph proteins. **(C)** Schematic representation of alternative open reading frames of MHV68 gL. Alternative start codons are indicated by arrowheads, SP: predicted signal peptide. Pairwise precipitation of soluble recombinant MHV68 gH ectodomain in complex with expression

bioRxiv preprint doi: https://doi.org/10.1101/2025.06.05.657996; this version posted June 5, 2025. The copyright holder for this preprint (which was not certified by peer review) is the author/funder. This article is a US Government work. It is not subject to copyright under 17 USC 105 and is also made available for use under a CC0 license.

constructs for full-length MHV68 gL (gH/gL) or MHV68 gL$^{\Delta N36}$ (37-173) (based on NP_044884.3) with murine EphA4. MHV68 gH alone was used as control. Precipitates were analyzed by immunoblot with indicated antibodies. **(D)** Protein accession numbers and percentage of identical amino acids in aligned regions of human and murine Eph proteins as determined by BLAST (Basic Local Alignment Search Tool). **(E)** Pairwise precipitation of soluble recombinant gH-FcStrep with individual murine Eph proteins. Precipitates were analyzed by immunoblot with indicated antibodies. For A, B, C, E, molecular weight is indicated in kDa.

bioRxiv preprint doi: https://doi.org/10.1101/2025.06.05.657996; this version posted June 5, 2025. The copyright holder for this preprint (which was not certified by peer review) is the author/funder. This article is a US Government work. It is not subject to copyright under 17 USC 105 and is also made available for use under a CC0 license.

S2 Fig

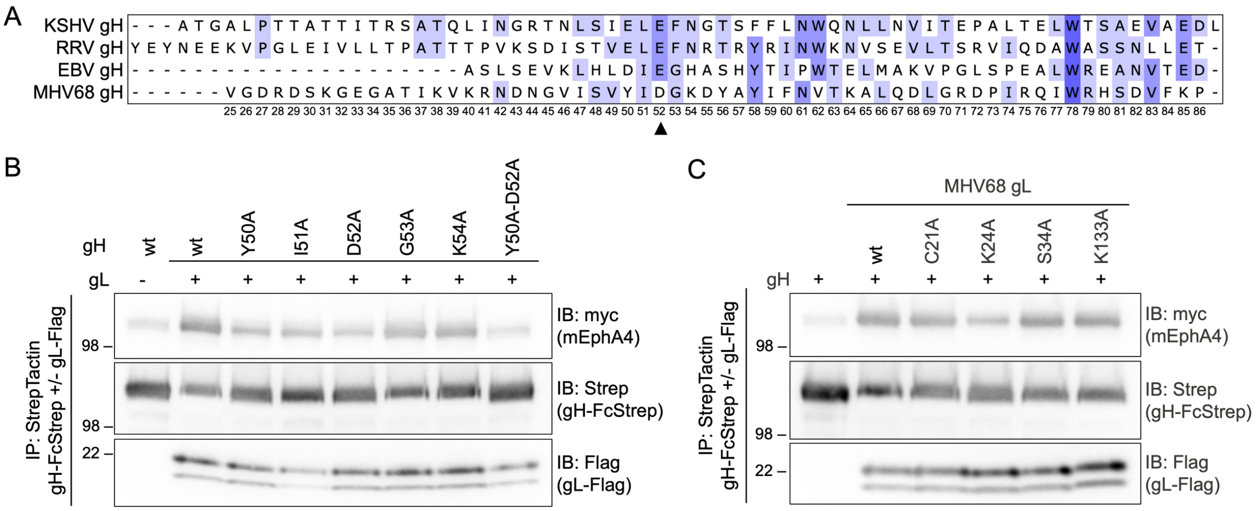

S2 Fig. Multiple residues on gH and gL mediate the MVH68 gH/gL Eph interaction.

(A) Multiple sequence alignment of gH D-I of human (KSHV [QFU18817], EBV [AIM62235]), rhesus macaque (RRV 26-95 [AAF60000]) and murine (MHV68 [NP044860]) GHVs. Numbers according to MHV68 gH. Arrowhead indicates R106$^{EphA4/EphB3}$ interacting residue D52$^{MHV68\ gH}$. **(B)** Single amino acids in the putative Eph interacting region in MHV68 gH were mutated to alanine. MHV68 gH^{ecto} mutants in complex with MHV68 gL were precipitated with murine EphA4. MHV68 gH-FcStrep was used as control. Precipitates were analyzed by immunoblot with indicated antibodies. **(C)** Putative Eph-interacting residues in MHV68 gL were mutated to alanine. MHV68 gH^{ecto} in complex with MHV68 gL mutants was precipitation with murine EphA4. MHV68 gH-FcStrep was used as control. Precipitates were analyzed by immunoblot with indicated antibodies. For B, C, molecular weight is indicated in kDa.

bioRxiv preprint doi: https://doi.org/10.1101/2025.06.05.657996; this version posted June 5, 2025. The copyright holder for this preprint (which was not certified by peer review) is the author/funder. This article is a US Government work. It is not subject to copyright under 17 USC 105 and is also made available for use under a CC0 license.

S3 Fig

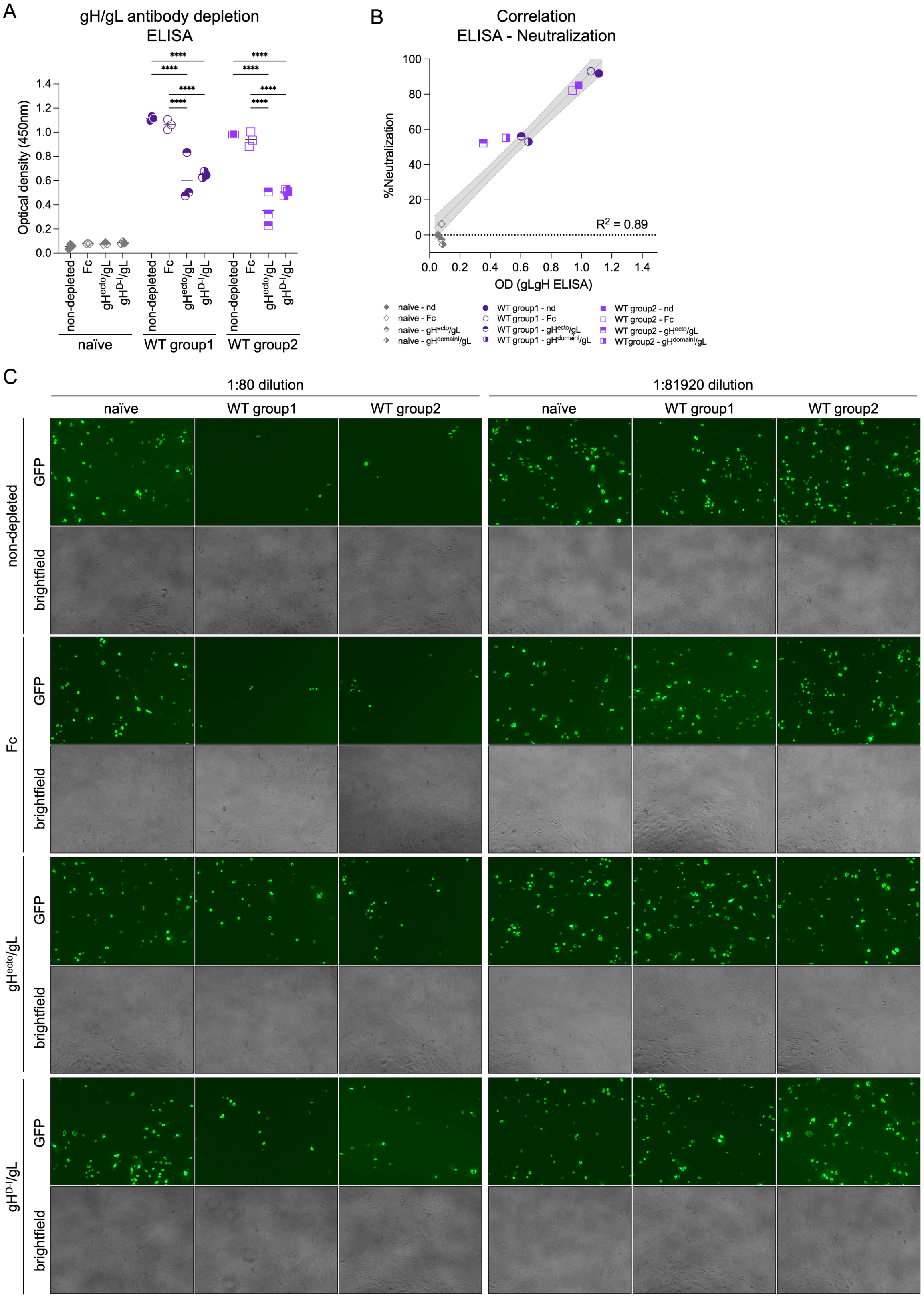

S3 Fig. Neutralizing antibodies in MHV68 infected mice target the MHV68 gH/gL complex.

bioRxiv preprint doi: https://doi.org/10.1101/2025.06.05.657996; this version posted June 5, 2025. The copyright holder for this preprint (which was not certified by peer review) is the author/funder. This article is a US Government work. It is not subject to copyright under 17 USC 105 and is also made available for use under a CC0 license.

(A) Antibodies to gH^{ecto}/gL or gH^{D-I}/gL were depleted using soluble complexes pre-coupled to magnetic beads. Fc was used as control. gH/gL-specific IgG from naïve or MHV68-infected C57BL/6 before and after adsorption was measured by MHV68 gL-gH ELISA. Background corrected optical density at 450 nm is shown. Mean and symbols representing individual experiments are shown. **(B)** Correlation of mean neutralization and optic density from three independent experiments. **(C)** Serum neutralization of MHV68 ORF59-GFP infection on NIH 3T3 cells is mediated by gH/gL-targeting antibodies. Antibodies to gH^{ecto}/gL or gH^{D-I}/gL were depleted using soluble complexes pre-coupled to magnetic beads. Fc was used as control. Micrographs were taken at 16 hpi. Statistical significance was evaluated by ordinary two-way ANOVA followed by Tukey's multiple comparisons test. ***: p-value < 0.001, ****: p-value < 0.0001.
